# Improving Procedural Documentation of Newly Diagnosed Pediatric Inflammatory Bowel Disease Patients: A Single-center Quality Improvement Study

**DOI:** 10.1097/pq9.0000000000000819

**Published:** 2025-06-04

**Authors:** Hamza Hassan Khan, Jordan S. Whatley, Carmine Suppa

**Affiliations:** From the * Division of Pediatric Gastroenterology, Department of Pediatrics, Medical University of South Carolina, Charleston, S.C.; † Division of Pediatric Gastroenterology, Department of Pediatrics, University of Arkansas for Medical Sciences, Little Rock, Ark.

## Abstract

**Introduction:**

Inflammatory bowel disease (IBD), including ulcerative colitis and Crohn disease (CD), presents significant challenges in management, particularly regarding standardized endoscopic scoring. This study aimed to assess and improve procedural documentation practices among endoscopists managing newly diagnosed pediatric IBD (PIBD).

**Methods:**

This quality improvement project involved a preintervention review of records for newly diagnosed patients with PIBD from January 2022 to December 2022 and a postintervention review of records from March 2023 to March 2024. We evaluated procedural documentation practices pre- and postintervention using control charts. We conducted an educational session on standardized procedural documentation for endoscopists in March 2023. Standardized procedural documentation was defined as the Mayo endoscopic score for ulcerative colitis and the simple endoscopic score for CD. We displayed a reminder flow diagram on the computer used by endoscopists for their procedural documentation.

**Results:**

In the preintervention period (n = 29), endoscopists used standardized documentation in 21% of cases (6/29). Postintervention (n = 43), standardized documentation use increased to 72% (31/43), demonstrating a 51% improvement. Subgroup analysis revealed variable adoption rates, with 100% for IBD-undetermined and 0% for patients with very early onset IBD. Control p-chart revealed a downward trend in the defect rate in the later months, suggesting improved adherence.

**Conclusions:**

Our initiative significantly enhanced the utilization of standardized endoscopic documentation among endoscopists for newly diagnosed patients with PIBD. This improvement underscores the effectiveness of structured educational strategies in promoting adherence to best practices. Future efforts should focus on sustaining these gains and addressing subgroup-specific challenges to optimize patient care in IBD management.

## INTRODUCTION

Inflammatory bowel disease (IBD), comprising both ulcerative colitis (UC) and Crohn disease (CD), is a chronic inflammatory condition of the gastrointestinal tract.^1^ CD involves any part of the gastrointestinal tract from the mouth to the anus, whereas UC typically involves only the colon.^2^ IBD-undetermined (IBD-U) is characterized by clinical, endoscopic, and histological findings of chronic colitis with subtle findings of CD and UC, but without specific features.^3^ Very early onset IBD (VEO-IBD) is defined as the diagnosis of IBD before 6 years of age.^4^

Complications of untreated IBD include intestinal strictures or stenosis, obstruction, fistula tracts, intra-abdominal abscesses, and dysplasia or malignancy.^5^ Endoscopic evaluation with biopsy is the standard of care for diagnosing IBD.^1^ Per the expert consensus recommendations of the Selecting Therapeutic Targets in Inflammatory Bowel Disease initiative of the International Organization for the study of IBDs, mucosal healing on follow-up endoscopic evaluation is the treatment target for both CD and UC.^6^ Standardized endoscopic severity scoring developed because of the endoscopists’ variability in subjective visual assessment of the mucosal disease activity.^5^ Selecting Therapeutic Targets in Inflammatory Bowel Disease-II recommends using the simple endoscopic score for CD (SES-CD), and the Mayo endoscopic score or the ulcerative colitis endoscopic index of severity for UC.^6^

We observed that interpreting the mucosal disease activity of patients diagnosed elsewhere but referred to our center for management was difficult when descriptive rather than standardized endoscopic documentation was used. In addition, we also observed that most of the endoscopists at our institution were not using standardized endoscopy scoring, particularly for newly diagnosed IBD patients. This quality improvement (QI) study aimed to identify our endoscopists’ current procedural documentation practices and implement strategies to improve the use of the standardized scores.

## METHODS

We completed this single-institution study at the Medical University of South Carolina Shawn Jenkins Children’s Hospital, a large tertiary medical center. The Division of Pediatric Gastroenterology, Hepatology and Nutrition completes approximately 1,100 endoscopies annually, making approximately 40 new pediatric IBD (PIBD) diagnoses yearly. The institution’s institutional review board approved this study as a QI project. The study included all patients newly diagnosed with PIBD between January 2022 and December 2022, as well as March 2023 and March 2024.

**Fig. 1.**
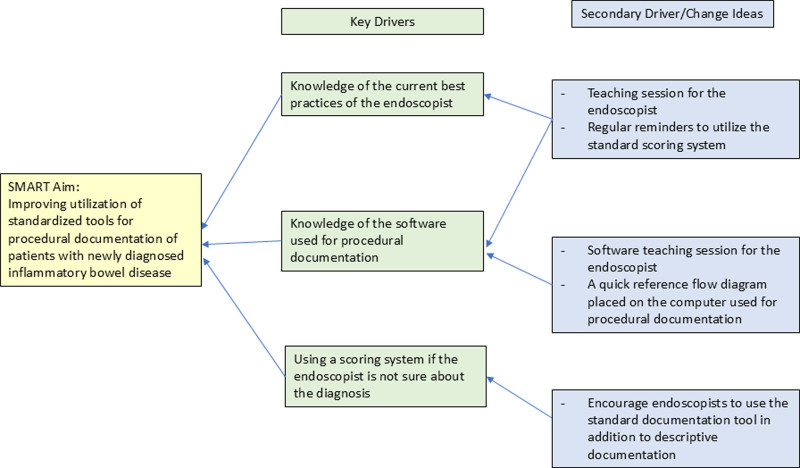
Key driver diagram for improving procedural documentation of newly diagnosed patients with PIBD.

To assess existing procedural documentation practices, we reviewed records of newly diagnosed patients with PIBD from our institutional registry from January 2022 to December 2022. This review process took place during January 2023 and February 2023; hence, data for those months were not included in this study. We extracted and reviewed the procedural documentation for these patients, focusing on the date of the procedure, the diagnosis, and the type of procedural documentation (descriptive versus standardized). Data were collected using Microsoft Excel 365 to evaluate the existing procedural documentation practices of the endoscopists. Figure 1 summarizes the key driver diagram, including primary and secondary drivers.

**Fig. 2.**
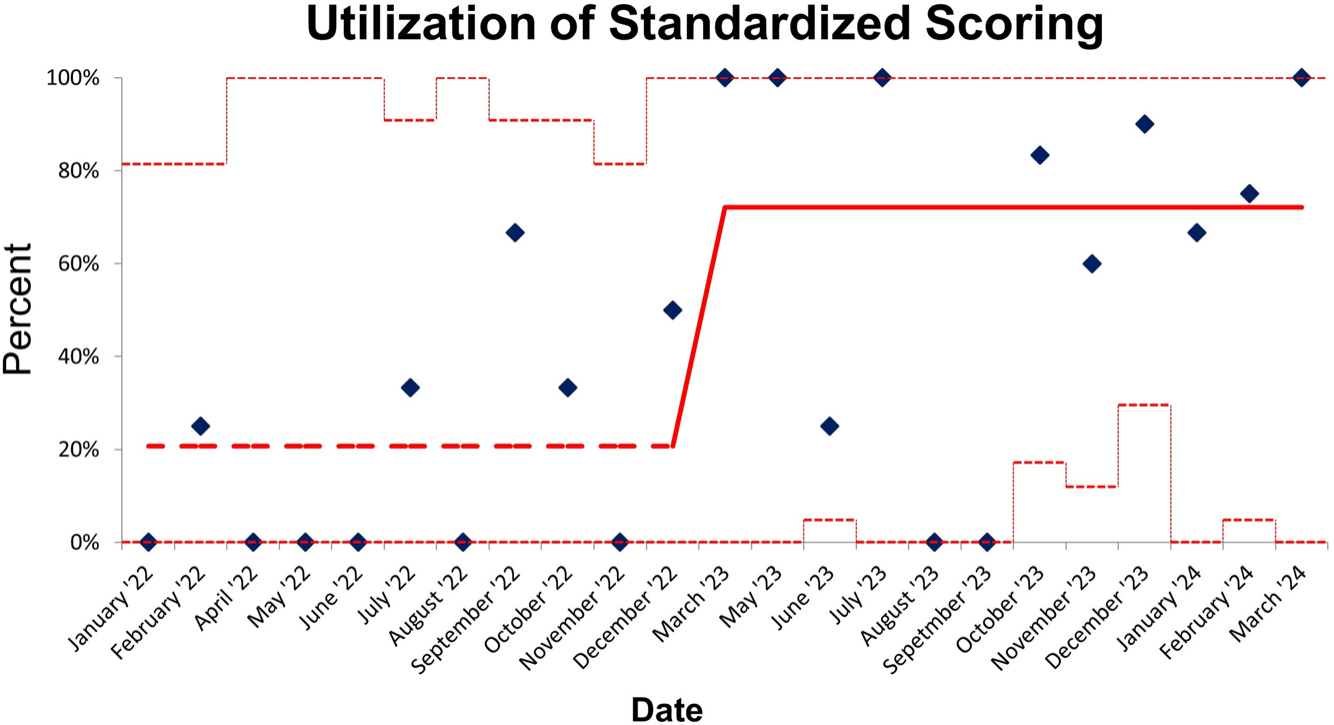
Control p-chart—utilization of standardized scoring before and after intervention in newly diagnosed patients with PIBD.

In March 2023, we conducted a teaching session for all endoscopists, consisting of 8 pediatric gastroenterologists and 3 pediatric gastroenterology fellows. This session covered the current endoscopy documentation practices, the objectives of our QI project, and a step-by-step guide to standardized documentation using our endoscopy documentation software, Provation (Provation Software, Inc., Minneapolis, Minn.). We created a flow diagram to remind endoscopists and assist them in navigating the Provation software to find the standardized documentation tools. (**See figure 1, Supplemental Digital Content 1**, which describes flow diagram to remind and assist endoscopists in navigating the Provation software to find the standardized documentation tools, https://links.lww.com/PQ9/A673.). We displayed a flow diagram on the computer in the endoscopy suite, which endoscopists use to document their procedures. Additionally, we sent 3 monthly reminder emails, including the flow diagram, to the endoscopists to encourage and remind the use of the standardized documentation tools for newly diagnosed patients with PIBD. Because there are no standardized documentation tools for VEO-IBD and IBD-U, endoscopists were required to use either SES-CD or Mayo score based on their best judgment of the endoscopic findings.^6^ (**See figure 2, Supplemental Digital Content 2**, which describes Mayo endoscopic score for UC, https://links.lww.com/PQ9/A674.) (**See figure 3, Supplemental Digital Content 3**, which describes SES-CD, https://links.lww.com/PQ9/A675.)

In April 2024, we collected data for the postintervention period (March 2023 to March 2024). We analyzed the data using frequency distributions, which consisted of categorical variables. We calculated descriptive statistics to assess differences between pre- and postintervention procedural documentation practices. P-chart was constructed using Microsoft Excel. Defects were defined as not utilitizing standardized documentation tools for newly diagnosed patients with PIBD. The authors would like to note that no endoscopist or author involved in this study has any financial ties to Provation Software, Inc.

## RESULTS

We included 29 newly diagnosed patients with PIBD in the institutional registry in the preintervention period. Endoscopists used standardized documentation tools in 6 of 29 (21%) patients. The use of standardized documentation varied among different PIBD subtypes: 0 (0%) of 2 IBD-U patients, 1 (50%) of 2 patients with VEO-IBD, 3 (30%) of 10 patients with UC, and 2 (13%) of 15 patients with CD.

We included 43 newly diagnosed patients with PIBD in the registry postintervention period. Standardized documentation tools were used in 31 (72%) of 43 patients. In the subset of the newly diagnosed patients with PIBD in the postintervention period, endoscopists used standardized documentation in 9 (69%) of 13 patients with UC, 21 (75%) of 28 patients with CD, 1 (100%) of 1 patients with IBD-U, and 0 (0%) of 1 patients with VEO-IBD. Of note, 3 colonoscopies were endoscopically normal in the postintervention period but histologically consistent with IBD, and 1 patient had poor bowel preparation; hence, endoscopists did not use standardized documentation in those instances. In addition, 2 endoscopists did not use standardized scoring in each of 3 instances of missed opportunities. In addition, in both preintervention and postintervention groups, if the standardized documentation tools were used, all the components of the tool were filled out, and there were no missing elements. Figure 2 demonstrates a control p-chart of standardized scoring utilization with high initial defect rates (low compliance) and a decline in defect rates over time (improvement in compliance). Utilization of standardized tools improved by 51% in the postintervention period compared with the preintervention period.

## DISCUSSION

Our QI study highlights significant improvement in using standardized endoscopic procedural documentation for newly diagnosed patients with PIBD following an educational intervention with visual reminders. Our initial review confirmed that only 21% of endoscopic procedures utilized the standardized tools before the intervention. However, following targeted education and reminders, we observed a remarkable increase to 72% in postintervention. This increase was also demonstrated in the control charts, showing a decrease in the rate of defects, which was defined as not utilitizing standardized documentation tools for newly diagnosed patients with PIBD, and improvement in compliance. The control p-chart demonstrates the process to be in statistical control. The high initial defect rate and low compliance point to a need for continued improvement. The observed decline in the defect rates and improvement in compliance over time reflects the positive effects of the interventions. This 51% marked improvement emphasizes the effectiveness of structured educational strategies in promoting adherence to best practices among endoscopists.^6^

For several reasons, improving the procedural documentation of newly diagnosed pediatric patients with IBD using standardized endoscopic scoring systems such as the SES-CD and the Mayo score is crucial. First, endoscopic assessment is the gold standard for evaluating mucosal disease activity, prognosis, and response to therapy in IBD. The use of standardized scoring systems such as SES-CD and the Mayo score ensures uniformity and objectivity in reporting mucosal appearance, which is essential for accurate clinical decision-making.^7^

Second, treating-to-target, which involves early intervention to prevent or limit intestinal injury, relies heavily on objective measures of disease activity. Endoscopic remission, defined as mucosal healing, is a key therapeutic goal in IBD management. Utilizing validated endoscopic scoring systems helps in achieving this goal by providing consistent and reliable measures of disease activity.^7^

Moreover, QI initiatives in IBD care, such as those implemented by the American Gastroenterological Association and the Crohn’s and Colitis Foundation of America, emphasize the importance of rigorous documentation, analysis, and feedback to improve patient outcomes. Improved documentation through standardized scoring systems can lead to better monitoring and management of the disease, ultimately enhancing the quality of life for patients with PIBD.^8,9^

The Simplified Endoscopic Mucosal Assessment for CD (SEMA-CD) has been developed as a novel method for assessing disease activity. The SEMA-CD is designed to be easy to use in routine clinical practice. It has shown a strong correlation with SES-CD, making it a reliable and reproducible tool for endoscopic assessment.^10^ The SEMA-CD is particularly advantageous due to its ease of use and sensitivity to change, which can further enhance documentation quality and patient care in clinical settings.^10,11^

The variability in the application of standardized documentation before the intervention may reflect several factors, including a lack of awareness, training, or the perceived complexity of using standardized scores. Also, some providers may be hesitant to use standardized IBD documentation based upon endoscopic evaluation alone before histological confirmation of the diagnosis, as there are more patients with colonic CD in the pediatric population who providers may initially mislabel as UC. Inconsistent documentation practices can lead to discrepancies in patient care and outcomes, particularly under chronic conditions such as IBD, where early and accurate assessment of mucosal involvement is critical for treatment optimization.^6^ The substantial improvement postintervention suggests that a focused approach to education, coupled with visual aids and ongoing reminders, can significantly enhance adherence to evidence-based practices. Of note, none of the endoscopists voluntarily reported increased documentation time using the standardized documentation. However, no formal feedback was collected for this study. Notably, although the overall increase in standardized scoring was significant, certain subgroups demonstrated variable uptake. For instance, endoscopists used standardized documentation in 100% of patients with IBD-U, whereas the VEO-IBD group did not see any standardized documentation used by endoscopists. This difference highlights a potential area for further targeted intervention, as these patients may present unique challenges, including categorizing them as CD or UC, requiring additional educational efforts or tailored documentation strategies.

Moreover, the postintervention data indicate that although standardized documentation for patients with UC (69.2%) and CD (75%) improved, it still leaves room for improvement because 28% of missed opportunities remain. Monitoring and feedback are essential to ensure that endoscopists sustain these improvements over time. Implementing ongoing educational sessions, refresher courses, and feedback mechanisms may further solidify the integration of standardized tools into everyday practice.

Our study emphasizes the importance of regular auditing and feedback in clinical practice. By systematically reviewing documentation practices, we can identify trends, address gaps, and continuously adapt our strategies to promote high-quality care. In a similar initiative, Shukla-Udawatta et al^12^ reported improving delivery of care through standardized monitoring in children with eosinophilic esophagitis, and Feldman et al^13^ improved the efficiency of their pediatric gastroenterology procedure suite by increasing procedure volume by 25%, on-time start improvement by 36%, decreasing turnover time by 34%, and decreasing postanesthesia care unit length of stay by 15%.

Limitations of our study include the single-center design limited to only newly diagnosed patients with PIBD, a relatively small sample size, variability in the endoscopist experience, 3 endoscopically normal colonoscopies for which standardized documentation was not used, and a limited 1-year postintervention follow-up period to assess the sustainability of the improvements.

Our QI initiative successfully increased the use of standardized endoscopic scoring tools among endoscopists managing newly diagnosed PIBD patients. This improvement is crucial for ensuring accurate assessments and facilitating mucosal healing, a key treatment target in IBD management. Future efforts should focus on sustaining these gains, addressing subgroup-specific challenges, and fostering a culture of QI in procedural documentation practices within our institution. We hope that our study will encourage other institutions to consider auditing their endoscopists’ procedural documentation and taking steps toward improving the standardized scoring system utility. By doing so, we aim to enhance the overall care and outcomes for patients with PIBD.

## Supplementary Material


