# Supplementary material for: Improving Procedural Documentation of Newly Diagnosed Pediatric Inflammatory Bowel Disease Patients: A Single-center Quality Improvement Study
Source: Pediatr Qual Saf. 2025 Jun 4;10(3):e819. doi: 10.1097/pq9.0000000000000819 (PMC12136661; doi:10.1097/pq9.0000000000000819)
Supplement: Supplementary file 1 [file pqs-10-e819-s001.pdf]

Quick Reminder

- **Simple Endoscopic Score for Crohn's Disease**
  - Findings → Colon → Inflammatory Bowel Disease → Crohn's Simple Endo Score

- **Mayo score for Ulcerative Colitis**
  - Findings → Colon → Inflammatory Bowel Disease → UC-Mayo Endo score
