# Supplementary material for: Improving Procedural Documentation of Newly Diagnosed Pediatric Inflammatory Bowel Disease Patients: A Single-center Quality Improvement Study
Source: Pediatr Qual Saf. 2025 Jun 4;10(3):e819. doi: 10.1097/pq9.0000000000000819 (PMC12136661; doi:10.1097/pq9.0000000000000819)
Supplement: Supplementary file 2 [file pqs-10-e819-s002.pdf]

Score	Disease activity	Endoscopic features
0	Normal or inactive	None
1	Mild	Erythema, decreased vascular pattern, mild friability
2	Moderate	Marked erythema, absent vascular pattern, friability, erosions
3	Severe	Spontaneous bleeding, ulceration
