# Supplementary material for: Improving Procedural Documentation of Newly Diagnosed Pediatric Inflammatory Bowel Disease Patients: A Single-center Quality Improvement Study
Source: Pediatr Qual Saf. 2025 Jun 4;10(3):e819. doi: 10.1097/pq9.0000000000000819 (PMC12136661; doi:10.1097/pq9.0000000000000819)
Supplement: Supplementary file 3 [file pqs-10-e819-s003.pdf]

	Simple endoscopic score			
**Variable**	**0**	**1**	**2**	**3**
Size of ulcers	None	Aphthous ulcers	Large ulcers	Very large ulcers
Diameter of ulcers	None	0.1–0.5 cm	0.5–2 cm	>2 cm
Ulcerated surface	None	<10%	10–30%	>30%
Affected surface	Unaffected segment	<50%	50–75%	>75%
Narrow-ings	None	Single, can be passed	Multiple, can be passed	Cannot be passed
